# Measurement precision of the disability for back pain scale-by applying Rasch analysis

**DOI:** 10.1186/1477-7525-11-119

**Published:** 2013-07-16

**Authors:** Yen-Mou Lu, Yuh-Yih Wu, Ching-Lin Hsieh, Chih-Lung Lin, Shiuh-Lin Hwang, Kuang-I Cheng, Yi-Jing Lue

**Affiliations:** 1 Department of Orthopaedics, School of Medicine, College of Medicine, Kaohsiung Medical University, Kaohsiung, Taiwan; 2 Department of Orthopaedics, Kaohsiung Medical University Hospital, Kaohsiung Medical University, Kaohsiung, Taiwan; 3 Department of Special Education, National Kaohsiung Normal University, Kaohsiung, Taiwan; 4 School of Occupational Therapy, College of Medicine, National Taiwan University, Taipei, Taiwan; 5 Department of Physical Medicine and Rehabilitation, National Taiwan University Hospital, Taipei, Taiwan; 6 Division of Neurosurgery, Department of Surgery, Kaohsiung Medical University Hospital, Kaohsiung Medical University, Kaohsiung, Taiwan; 7 School of Medicine, College of Medicine, Kaohsiung Medical University, Kaohsiung, Taiwan; 8 Department of Anesthesiology, Kaohsiung Medical University Hospital, Kaohsiung Medical University, Kaohsiung, Taiwan; 9 Department of Physical Therapy, College of Health Science, Kaohsiung Medical University, Kaohsiung, Taiwan; 10 Neurology and Master’s Program in Neurology, School of Medicine, College of Medicine, Kaohsiung Medical University, Kaohsiung, Taiwan; 11 Department of Physical Medicine and Rehabilitation, Kaohsiung Medical University Hospital, Kaohsiung Medical University, Kaohsiung, Taiwan

**Keywords:** Back pain, Rasch analysis, Oswestry disability index, Functional measure, Disability

## Abstract

**Background:**

The Oswestry Disability Index (ODI) is widely used for patients with back pain. However, few studies have examined its psychometric properties using modern measurement theory. The purpose of this study was to investigate the psychometric properties of the ODI in patients with back pain using Rasch analysis.

**Methods:**

A total of 408 patients with back pain participated in this cross-sectional study. Patients were recruited from the orthopedic, neurosurgery, rehabilitation departments and pain clinic of two hospitals. Rasch analysis was used to examine the Chinese version of ODI 2.1 for unidimensionality, item difficulty, category function, differential item functioning, and test information.

**Results:**

The fit statistics showed 10 items of the ODI fitted the model’s expectation as a unidimensional scale. The ODI measured the different levels of functional limitation without skewing toward the lower or higher levels of disability. No significant ceiling and floor effects and gaps among the items were found. The reliability was high and the test information curve demonstrated precise dysfunction estimation.

**Conclusions:**

Our results showed that the ODI is a unidimensional questionnaire with high reliability. The ODI can precisely estimate the level of dysfunction, and the item difficulty of the ODI matches the person ability. For clinical application, using logits scores could precisely represent the disability level, and using the item difficulty could help clinicians design progressive programs for patients with back pain.

## Background

Back pain is a common health problem and one of the most costly conditions in many countries [1]. Thus, it is essential to use a precise tool to assess pain and disability. The Oswestry Disability Index (ODI) is one of the most widely used disease-specific self-administered questionnaires for measuring back pain [2,3]. The questionnaire assesses the pain problem and the resulting functional disability [4]. Strict examination of the psychometric properties of the ODI by modern measurement theory is needed for precise measurement of the level of functional limitation in back pain.

Compared with traditional classical test theory (CTT), the Rasch model overcomes the drawbacks of scoring. In CTT, the person’s ability and the difficulty of each item cannot be estimated separately; the score of each item in CTT is ranked as the same (on an ordinal-level scale), without considering differences in the difficulty of the individual question. Total scores are used in CTT to represent the latent traits of subjects without concern for differences in difficulty of individual items. However, strict measurement requires a linear (continuous-level) additive scale with equal units that allow the scale to be manipulated mathematically. In the Rasch model, the items included in an instrument must be defined as only a single construct (unidimensionality) [5]. When the instrument is non-unidimensional, some items would not measure the same trait; and other constructs might be measured. It is not appropriate to add all non-unidimensional item scores as a total score to represent the character of the trait [6]. The Rasch model uses the probability of a subject’s response to individual items as the latent traits. The Rasch model transforms ordinal scores into interval scores along the logit scale [7,8], which successfully ranks the item difficulty among items, thereby overcoming the scoring problems of CTT. Furthermore, the changes in the logit scale will be a more precise and valid indicator of changes in a client's ability than are the changes in an ordinal scale.

Only a few studies have used the Rasch model to examine psychometric properties of the ODI; however, sampling shortcomings do exist. For example, participants in these studies were recruited from one outpatient departments and included only patients with non-specific low back pain [9-11]. Overly homogenous samples contain some potential problems associated with investigating the psychometric properties for an outcome measure. If the sample is homogeneous, the correlation between the respective item and the total sum score will be lower, which will further influence the reliability and validity of the scale; and a restricted range of scores will likely attenuate the estimate [12,13]. When subjects are very similar, a narrow range of ability may create the incorrect conclusion that the scale poorly targets the person ability and is unable to discriminate between persons. Furthermore, the variance explained by the measure is small and ceiling/floor effects may exist. For example, when most participants possess similarly mild functional limitation characteristics, the scale may have a significant floor effect. On the other hand, it is only when severe cases are included that the scale will be induced to show a significant ceiling effect. Furthermore, an overly homogenous sample may demonstrate that the scale has difficulty discriminating such individuals. Including heterogenous sampling matches the various conditions of the patients and the wide range of the patients’ behaviors can then be observed. Therefore, an important next step is to examine the scale with diverse samples to determine if the structure of ODI will hold true [9-11]. In addition to the sampling problem, inconsistent results have also been found among studies, for example, that the pain item misfit and should be deleted was reported in Page et al. [10]; however, when Davidson et al. explored three versions of the ODI, they did not find that the pain item misfit [9]. Furthermore, the order of item difficulty differs among studies, for example, sexual activity was reported as less influenced by back pain among 10 items in Page et al.’s study [10]; however, other studies reported that sexual activity was easily influenced by back pain [9,11]. Therefore, these important psychometric properties of the ODI still exist in the questions.

In addition to the importance of unidimensionality and item difficulty, the Rasch model also provides many useful measurement properties, such as whether the response categories of each item are appropriate (category function), whether the items are equivalent in meaning to different respondents (differential item functioning, DIF) and how precisely the disability of patients with various ability levels can be estimated (reliability and test information function). The purpose of our study therefore, was to re-examine the psychometric properties of ODI with Rasch analysis by improving the sampling strategy in patients with back pain.

## Methods

### Participants

Participants with various types of back pain were recruited from Kaohsiung Medical University Hospital and Kaohsiung Municipal Hsiao-Kang Hospital of Taiwan from March 1, 2007 to December 31, 2009. The outpatients were recruited from the orthopedic, neurosurgery, rehabilitation departments, and pain clinic, and the inpatients were recruited from the orthopedic and neurosurgery departments. Patients were identified by physicians on the basis of symptoms, physical signs, and imaging study results. The criterion for inclusion in the study was “A patient with back pain with or without leg pain.” The diagnoses of back pain included spondylolisthesis, degenerative joint disease, herniated intervertebral disc, fracture, and nonspecific back pain. Patients suffering from other types of pain predominantly without back pain were excluded. We also excluded participants with cancer, rheumatic disease, psychological and cognitional problems, or pregnancy. The study was approved by the hospital’s Institutional Review Board (KMUH-IRB-970405, 980040), and written informed consent was obtained from all the participants.

### Procedure

After the diagnosis of back pain, each participant was asked to complete a questionnaire booklet, which contained the Chinese ODI 2.1 version [14], the Visual Analog Scale (VAS) [15], and demographic questions. For inpatients, the participants were assessed before the day of surgery. Elderly with presbyopia who complained about ocular discomfort while reading the questionnaire were interviewed face-to-face by a trained physical therapist.

### Instrument

The ODI contains 10 items that measure the degree to which back or leg trouble has affected the ability to manage activities of everyday life [16]. The 10 items ask about the following: pain intensity (ODI 1), the level of disability of personal care (ODI 2); lifting (ODI 3); walking (ODI 4); sitting (ODI 5); standing (ODI 6); sleeping (ODI 7); sex life (ODI 8); social life (ODI 9); and traveling (ODI 10). Each item is scored on a 6-point scale, with 0 representing no limitation, and 5 representing maximal limitation. The range of the ODI raw score (the sum of the scores from the 10 items) is from 0 to 50. The Chinese ODI 2.1 is a well developed questionnaire following the guidelines of cross-cultural adaptation procedures, using simple wording at an elementary school level. The questionnaire has been verified with satisfactory test-retest reliability and convergent and divergent validity [14,17].

### Statistical analyses

Winsteps^©^ Rasch analysis software using the partial credit model for polytomous items was used for Rasch analysis [18,19]. The Student’s *t-*test (SPSS software) was used to compare the differences in pain severity (VAS) and functional limitation (ODI) between inpatients and outpatients [20].

#### Unidimensionality

The infit and outfit statistics were used to inspect whether the data fit the model’s expectations. Infit statistics are sensitive to unexpected behavior of the patient’s responses on items near the patient’s measure level, and outfit statistics are sensitive to unexpected behavior far from the person’s measure level [8,21]. The value of weighted mean square of infit mean square (InfitMNSQ) and unweighted mean square of outfit mean square (OutfitMNSQ) were used as the fit indicators of the model. The acceptable ranges of both the values of the InfitMNSQ and OutfitMNSQ are between 0.5 and 1.5 [22].

Unidimensionality was also examined with principal component analysis of the standardized residuals (PCA residuals); the variance explained by measures should be large, while unexplained variance (the residual) in the first contrast should be small. The criterion level for the unidimensionality assumption of the variance explained by measures should be over 50% and the eigenvalue of unexplained variance explained by first contrast should be smaller than 2 [22].

#### Item difficulty and targeting

To identify the level of challenge for a patient performing the activities that were designed from ODI, the item difficulty was examined. Targeting was used to examine how well the distribution of ODI measured the disability consistent with the levels of functional limitation of the patients [23]. The item difficulty was calculated and expressed as a logit in Rasch analysis. Besides the item difficulty, the person-item response threshold location was plotted to assess the targeting and notable gaps of the item response thresholds.

#### Category function

To determine whether the response categories were appropriate, the category function was examined by assessing the plot of the probability category curves for each item of ODI. Each curve corresponds to the difficulty (x-axis) and the probability (y-axis) of one response category. The step difficulty (threshold) is the intersections of successive category probability curves. To have a satisfactory response category design, the order of the step difficulties should be a monotonic progression. [6,24,25]. Disordering of the step difficulties is defined as the difficulty of the higher- numbered step being lower than its adjacent lower- numbered step. If disordering of the step difficulties occurs, this indicates that the response categories need to be adjusted.

#### DIF

To examine whether the item manifests a different level of difficulty for different groups, the statistical method of DIF was used, meaning that some other factors or different latent traits may exist between groups that have influenced the item response. The Educational Testing Service (ETS) uses Delta units to assess the degree of item DIF: A Delta units smaller than 1 represents a negligible item DIF, between 1.0-1.5 (absolute DIF contrast 0.43-0.64 logits) indicates a slight-to-moderate item DIF, and larger than 1.5 is a moderate-to-large item DIF [26]. We assessed the DIF for different age groups (<65 and ≧65 years) and by gender.

#### Reliability and test information function

The reliability of the ODI was examined by the person reliability. A reliability coefficient equal to 0.7 or higher is considered as adequate for group comparisons [27]. The separation coefficient was also calculated (separation coefficient = √(person reliability)/(1-person reliability)), and the value of 2 or higher is acceptable [22,28], and the separation coefficient can be used to calculate the number of distinct strata of persons. The number of persons that can be distinguished in the sample is calculated as 4 times the separation coefficient plus 1, and then this figure is divided by 3 [29].

To identify the information demonstrated from the test, the test information curve was plotted. The inverse square root of the test information is the standard error (SE) of the Rasch person measure. Each individual item of information adds up to produce the test information. The curve provides the visual interpretation of test information plotted according to each person’s ability. The amount of information provided by a test could be referred to as the estimated precision [30]. The precision was defined as the SE smaller than 0.5, while the corresponding value of information and the reliability would then be > 4 and > 0.75, respectively [30].

## Results

### Demographic data and score distribution

The demographic data of the participants are shown in Table 1. A total of 408 patients with back pain participated in this study: 268 participants were outpatients and 140 were inpatients. Some of the outpatients (n=91) had undergone surgery (at least 3 months previously), and had recurrent back pain. The number of females (n=263) was nearly twice that of males (n=145). About 40% of participants were elderly, and about 15% needed the therapists’ help in completing the questionnaire. Most participants (about 80%) were in the chronic stage. The pain severity of the participants varied from mild to severe (VAS: 0.2-10). The disability level of the participants varied from minimal to bed-bound (ODI raw score: 1–50). Inpatients had more severe pain and more functional limitation than did outpatients (*p* = 0.001 and *p*<0.001 respectively).

**Table 1:** Demographic characteristics of the participants (n=408)

**Characteristic**	**Mean ± SD (range) percentage**
Age (years)	59.0±14.8 (15–86)
<35	8.5%
35–49	14.3%
50–64	36.0%
65–79	35.4%
80-86	5.8%
Gender	
Male	35.5%
Female	64.5%
Disease duration (month)	54.2±79.8 (0.3-600)
Acute (<6wk)	7.8%
Subacute (6wk-3mo)	12.0%
Chronic (>3mo)	80.2%
VAS score	5.2±2.8 (0.2-10)
Outpatients	5.3±2.5
Inpatients	6.2±2.4 (*p* = 0.001)
Education	
Elementary school	49.2%
Secondary school	31.2%
University	19.6%
Marital status	
Single/widow	23.1%
Married	76.9%
Occupation	
Office worker	10.9%
Laborer	17.8%
Housewife	37.8%
Student	2.2%
Retired	20.8%
Unemployment	10.5%
Diagnosis	
Spondylolisthesis	31.7%
Degenerative disc disease	17.4%
Herniated intervertebral disc	20.4%
Fracture	12.9%
Non-specific back pain	17.6%

### Unidimensionality

The results of InfitMNSQ and OutfitMNSQ showed that 10 items of ODI fitted the model’s expectations, indicating a unidimensional scale, with the values being within an acceptable range of 0.5-1.5 (Table 2) [22]. The results of PCA residual analysis showed that 57.2% of the variance was explained by measures, and the eigenvalue of unexplained variance explained by first contrast was only 1.7 (<2), which indicated good fit of a unidimensional model for ODI [22].

**Table 2:** Item difficulty and fit statistics of the 10 items of ODI

**Item**	**Difficulty logit**	**SE logit**	**Infit MNSQ**	**Outfit MNSQ**
ODI 2: Personal care	0.84	0.05	0.87	0.89
ODI 7: Sleeping	0.61	0.06	1.40	1.49
ODI 5: Sitting	0.34	0.05	1.29	1.39
ODI 4: Walking	0.26	0.05	0.77	0.75
ODI 1: Pain intensity	−0.10	0.06	1.26	1.21
ODI 9: Social life	−0.17	0.05	0.80	0.78
ODI 8: Sex life	−0.32	0.07	1.03	1.06
ODI 6: Standing	−0.48	0.05	0.79	0.75
ODI 10: Traveling	−0.48	0.05	0.74	0.71
ODI 3: Lifting	−0.49	0.05	1.14	1.20

### Item difficulty and targeting

**Figure 1:**
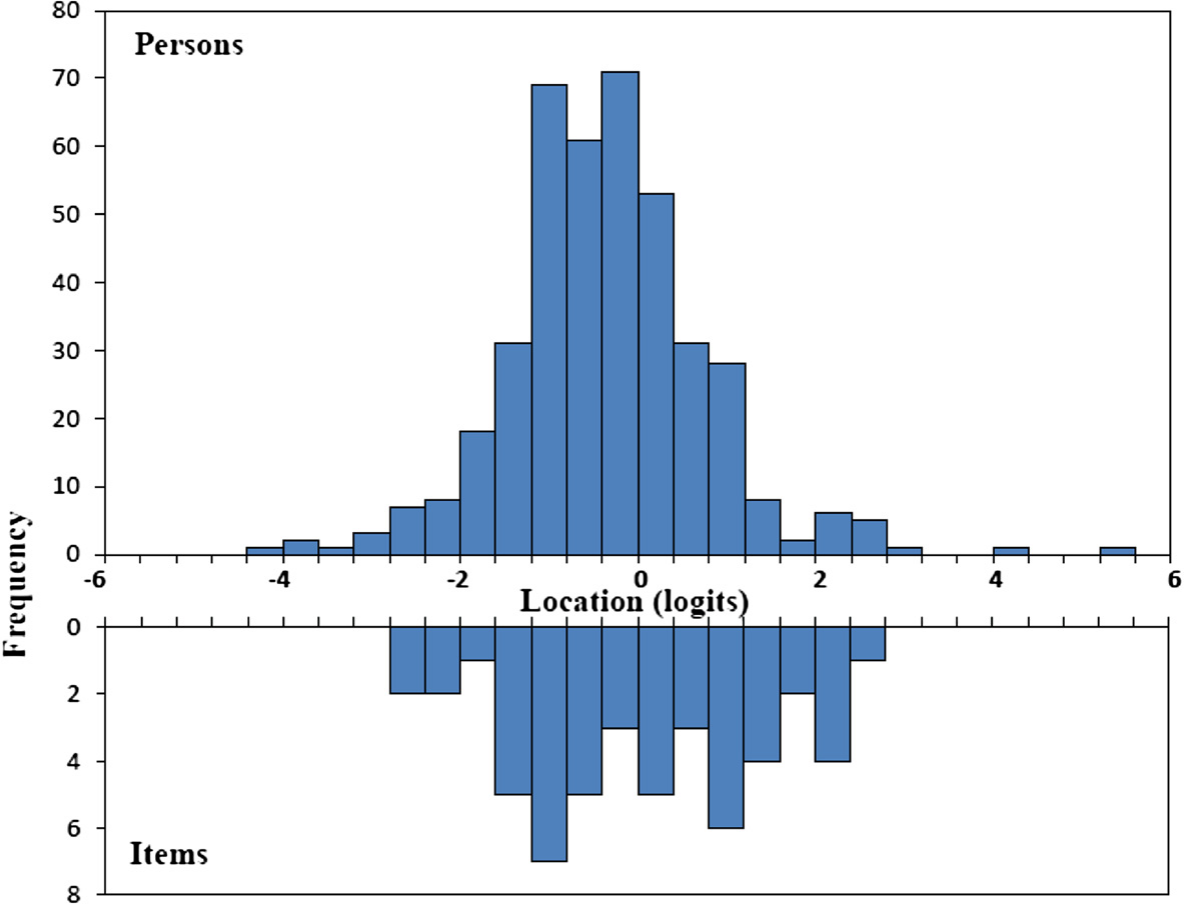
Distribution of patient reports of disability and item response thresholds locations (in logits).

The item difficulty of each item is represented in Table 2. The highest item difficulty measure (strong trait of disability) was item 2, which measured the disability of personal care. The lowest item difficulty measure (weak trait of disability) was item 3, measuring the disability of the lifting activity. The mean patient location (−.0.34±1.08 logits) was approximated to the mean item location (0.00±0.46 logits). Person-item response thresholds locations is shown in Figure 1; this represents the frequency distribution of the patients with different disabilities and the number of response categories of items distribution, to measure the different disabilities (in logit units). The range of the item response thresholds was 5.05 logits, which evenly covered the patient’s distribution; no gaps were reported.

### Category function

**Figure 2:**
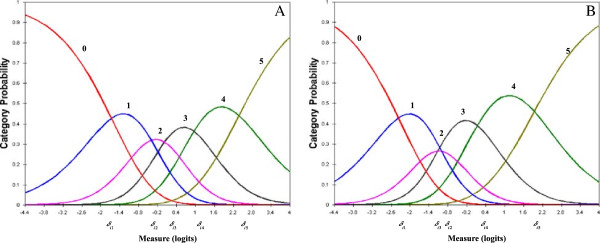
**The response category probability curve for a five-step item. (A)** An example of a well-functioning category response (item 4), showing a monotonic increase of the thresholds (***δ***) in the order of the logits level. **(B)** An example of a poorly-functioning category response (item 6), showing a disordering of the thresholds, and with the ordering of the ***δ***_***i*2**_ (categories between 2 and 3) and ***δ***_***i*3**_ (categories between 3 and 4) reversed.

Four items (items 4, 5, 7, and 9) had well-functioning responses, and the values of 5 step difficulties of each item are shown in Table 3. Figure 2-A demonstrates the category probability curves from item 4, an example of a well-functioning response scale with monotonic increase of the thresholds. Thresholds (*δ*) are located at the certain functional disability level for which pairs of successive responses are equally probable. The values of the thresholds (step difficulties) of *δ*_*i1*_ to *δ*_*i5*_ illustrated a monotonic increase in the thresholds. Figure 2-B is an example (item 6) of disorder category response respectively. The value of *δ*_*i2*_ was greater than *δ*_*i3*_ indicating disorder of the category response [6,22,25]. Six items (items 1, 2, 3, 6, 8 and 10) exhibited disordering of the step difficulties. The observed counts of each response category of disorder items were checked and then response categories of two adjacent items combined together (three adjacent response categories for item 3 were combined). The category function analysis was reapplied, and the step difficulties for each item showed a monotonic increase in the thresholds. The results of PCA residual analysis showed that 56.6% of the variance was explained by measures, and the eigenvalue of unexplained variance explained by first contrast was 1.8. The largest values of InfitMNSQ and OutfitMNSQ items still involved item 7 (these values were 1.5 and 1.59 respectively); other items were within acceptable ranges.

**Table 3:** Step difficulties represented with logit unit of the ODI with 6-level scaling

**Item**	***δ***_***i*1**_	***δ***_***i*2**_	***δ***_***i*3**_	***δ***_***i*4**_	***δ***_***i*5**_	Disordering of the step difficulties (*)			
						_**1–2**_	_**2–3**_	_**3–4**_	_**4–5**_
ODI 1: Pain intensity	−2.61	−1.34	0.99	0.86	1.62			*	
ODI 2: Personal care	−0.19	−0.62	0.96	2.01	2.02	*			
ODI 3: Lifting	−2.65	−0.16	−0.94	−1.01	2.30		*	*	
ODI 4: Walking	−1.67	−0.40	0.02	0.95	2.38				
ODI 5: Sitting	−1.08	−0.99	−0.01	1.36	2.40				
ODI 6: Standing	−2.36	−0.99	−1.12	0.14	1.94		*		
ODI 7: Sleeping	−1.35	0.82	0.91	1.33	1.35				
ODI 8: Sex life	−1.26	0.44	−1.34	0.16	0.39		*		
ODI 9: Social life	−1.48	−1.03	−0.47	0.67	1.46				
ODI 10: Traveling	−2.01	−0.72	0.39	−0.48	0.42			*	

*δ*, thresholds: the measure ability in the equally probable of two successive responses.

*δ*_*i*1_: indicates the threshold belonging to the first and the second responses.

### DIF

DIF was examined for age and gender of each item. For gender, the range of the absolute DIF contrast was 0 to 0.21 logits, equal to 0 to 0.49 Delta units. Negligible DIF existed for all items. For age, the range of the absolute DIF contrast was 0.07 to 0.49 logits, equal to 0.16 to 1.15 Delta units. Only the item concerning sleep (0.49 logits) with slight item DIF was observed.

### Reliability and test information function

The reliability coefficient of the 10 items was 0.89. The separation coefficient was 2.83, indicating that four levels ((4x2.83+1)/3=4.11) of person ability in this sample can be differentiated by the scale; these can be named as high, above average, below average, and low functional disability [29].

The mean value of item and person measure parameters were 0 (SD=0.46) and −0.34 (SD=1.08) logits respectively. The raw ODI scores and conversion to transformed logits scores and percentages of participants are shown in Table 4. No significant ceiling and floor effects were found.

**Table 4:** Raw score, logit score and standard error of the ODI

**Raw score**	**Logit score**	**Standard error**	**Number (%) of participants**	**Raw score**	**Logit score**	**Standard error**	**Number (%) of participants**
0	−5.39	1.85	0(0)	26	0.03	0.29	7(1.7)
1	−4.13	1.03	1(0.2)	27	0.12	0.30	9(2.2)
2	−3.37	0.75	3(0.7)	28	0.21	0.30	3(0.7)
3	−2.90	0.63	3(0.7)	29	0.30	0.30	17(4.2)
4	−2.56	0.55	7(1.7)	30	0.39	0.31	17(4.2)
5	−2.29	0.49	5(1.2)	31	0.49	0.31	13(3.2)
6	−2.07	0.45	5(1.2)	32	0.59	0.32	7(1.7)
7	−1.87	0.42	2(0.5)	33	0.69	0.32	7(1.7)
8	−1.71	0.40	7(1.7)	34	0.79	0.33	8(2.0)
9	−1.56	0.37	15(3.7)	35	0.90	0.33	11(2.7)
10	−1.43	0.36	9(2.2)	36	1.01	0.33	11(2.7)
11	−1.30	0.34	14(3.4)	37	1.13	0.34	2(0.5)
12	−1.19	0.33	13(3.2)	38	1.24	0.35	1(0.2)
13	−1.09	0.32	10(2.5)	39	1.37	0.36	4(1.0)
14	−0.99	0.31	17(4.2)	40	1.50	0.37	2(0.5)
15	−0.89	0.30	19(4.7)	41	1.64	0.38	1(0.2)
16	−0.80	0.30	11(2.7)	42	1.79	0.40	0(0)
17	−0.72	0.29	18(4.4)	43	1.96	0.42	2(0.5)
18	−0.63	0.29	9(2.2)	44	2.14	0.45	3(0.7)
19	−0.55	0.29	11(2.7)	45	2.36	0.48	3(0.7)
20	−0.47	0.29	22(5.4)	46	2.61	0.53	5(1.2)
21	−0.38	0.29	21(5.1)	47	2.93	0.60	1(0.2)
22	−0.30	0.29	6(1.5)	48	3.37	0.73	0(0)
23	−0.22	0.29	13(3.2)	49	4.10	1.02	1(0.2)
24	−0.14	0.29	14(3.4)	50	5.33	1.84	1(0.2)
25	−0.06	0.29	17(4.2)				

**Figure 3:**
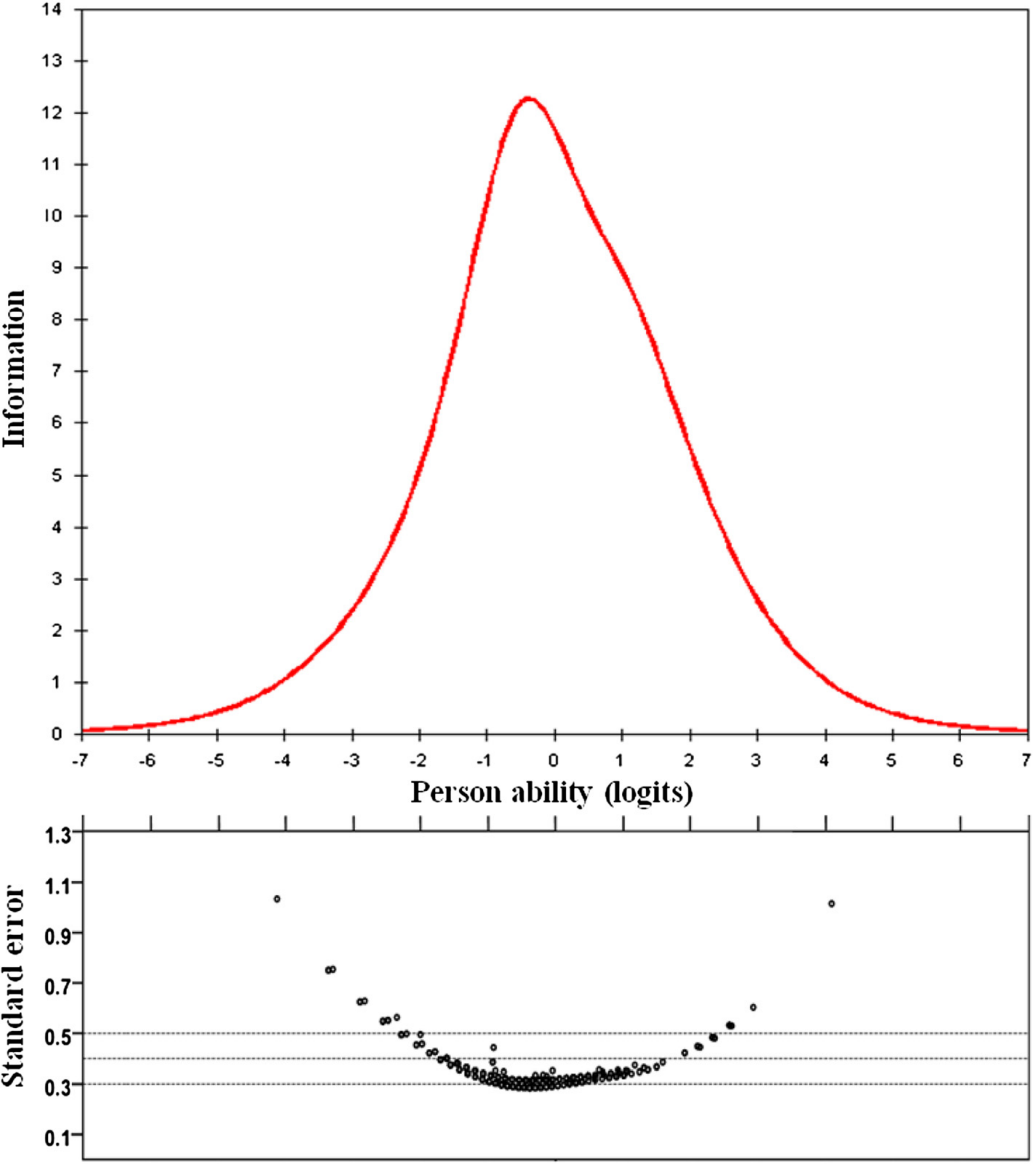
**The upper panel shows the test information curve.** A person with −0.34 logits measure offered maximum information of the dysfunction condition. In the lower panel, the standard error of person ability estimated to ODI 10-items from 408 patients.

The test information curve and SE according to person ability are shown in Figure 3. The shape of the test information curve was bell-shaped with its maximum at the middle of the person measure scale. The maximum information point was on −0.34 logit, so a person in the disability level (−0.34 logit) of the measure would provide the maximum information for disability due to back pain. The value of the maximum test information of our study was more than 12. The SE was small (≤ 0.5), with large information (≥ 4) when the person ability was from −2.3 to 2.3 logits. The ODI 0–50 raw scores, transformed logits scores and its SE are shown in Table 4. In a range of 5 to 45, the raw score equaled −2.29 to 2.36 logit score and the SE was small (≤ 0.5).

## Discussion

This study used modern measurement theory to examine unidimensionality and measurement properties of the ODI for assessing the disability level of patients with back pain. It is essential to precisely assess the disability to determine the amount of treatment effects before and after treatment for patients with back pain. The 10 items of ODI contributed a unidimensional construct measuring the different levels of functional limitation; the difficulties of these items were well targeted to the person ability without skewing toward the lower or higher levels of disability. The ODI is a reliable and precise measurement scale but exhibits response category disorders in some items.

The ODI items fit the model for measuring the functional limitation of activities. Compared with previous studies, item 1 (pain intensity) did not fit the model [10,11]. The reason for the difference might be due to using different versions of the test. In Page et al. and White et al.’s studies, the revised version was used. The pain intensity item asked about the issue of taking painkillers; however, version 2.1, used in our study did not ask about taking painkillers. Comparing the item fit among these items, the greatest values of infitMNSQ and outfitMNSQ was the sleeping item (Table 1). There are different interpretative guidelines to examine the item fit [22,31,32], the Winstep manual suggests the range of 0.5-1.5 as being productive for measurement, while Lai et al. used more strict criteria (range: 0.7-1.3). Deleting or retaining the borderline infitMNSQ/outfitMNSQ item may accord to the purpose of Rasch analysis. If the purpose of analysis is to develop or shorten the scale, item deletion can be used to construct a shortened scale. If the item provides useful clinical information, the scale is already developed and short enough, and the item need not be removed. For the sleeping issue, consistent evidence from many studies found that chronic low back pain was associated with greater sleep disturbance and the sleep problems should be addressed as an integral part of the pain management plan [33,34]. Because the ODI is short and widely used, and because sleep disturbance is an important issue for these patients, we do not suggest removing the item. The item on sleeping adds helpful information concerning the patient with back pain in clinical practice.

The order of item difficulty could be used as a reference of priority of progressive management program design according to the level of difficulties from with different activities (Table 2). The order of item difficulty for most items in our study was similar to that in other studies. The higher logit items (more disability) were personal care and sleeping, which were noted in individuals with the most severe levels of functional limitation. The lower logit items (less disability) were lifting and standing. For those with relatively low levels of physical limitation, lifting and standing were easily affected in patients with back pain. However, for the traveling item, the item difficulty was lower logit than other studies; it indicated the functional limitation was more prominent in Chinese for traveling. In Chinese culture, a person with back pain should decrease traveling activities as much as possible to avoid deteriorating back pain. Therefore, the activity involved in traveling may be thought of as a “difficulty” activity. The functional limitation of one’s sex life was also an important disability indicator for patients in our study; the item with a lower logits difficulty indicates that sex life is easily influenced by the presence of back pain. In addition to the activities just mentioned, in order to provide comprehensive care, clinicians also need to pay attention to problems in the patient’s social life, owing to the problems being also commonly influenced.

The precision of the test depends not only on the construction of the test, but also on the distribution of the sample of the patients being tested. Several indices were used to examine the targeting and precision of the test, such as comparing the mean person ability versus the mean item difficulty, inspecting the person-item response thresholds locations, checking the person reliability, standard error and the test information function. The results showed the targeting and precision of the test were good for ODI; the person-item response thresholds locations were also shown evenly spanned a similar range as for the persons (Figure 1). Davidson’s study included only ambulatory patients from physiotherapy departments, and the result showed most persons had lower logits difficulty (less disability), while items had higher logits difficulties (more disability) [9]. The more the mean person measure differs from zero, the more the set of items is mistargeted to the person ability. Back pain is a common reason for physical visits and hospitalizations in many clinical departments. For outpatient clinics, about 50% of patients are seen in the orthopedic department and 30% in rheumatology, pain, and neurosurgery departments [1,35]. For hospitalization, about 45% of back pain patients are found in orthopedic and neurosurgical wards [36]. We included both inpatients and outpatients from different departments of two hospitals, which may reduce the sampling bias, and the ODI scores showed no skewing toward the lower or higher levels of individual disability.

The test information curve provides more useful information, representing at the special point of ability of a person how precisely the test can estimate his/her disability. The reliability coefficient of this study was high (reliability coefficient=0.89) which was better than the previous studies (coefficient: 0.77-0.88); furthermore, our study showed the SE of each item was very small (Table 1). To our knowledge, this is the first study reporting the test information function for ODI. As shown in Figure 3, the ODI provides the greatest information when a person is at the −0.34 logits ability level. On the other hand, for a person with very low or very high logits ability level, the ODI is less reliable in measuring his/her disability level.

Two disadvantages of ODI were found by Rasch analysis: the category responses disorder in some items, and sleeping item with DIF. The category response of 6 of the items was not appropriate due to disordering of the step difficulties (Table 3). The finding is similar to that of other studies [9-11], and is also a common problem of a rating scale; too many category responses will easily disorder the step difficulties and some responses are not needed due to very low frequency for the subjects to choose [6,22]. The DIF analysis was used to differentiate whether the item was different between different groups of subjects, the sleeping item showed mild item bias between age groups, as disturbed sleeping due to pain was dominant in the younger patients. The reason for this is not clear, and needs further study. Collapsing some of the responses improves the scaling, and the DIF item may need to be deleted. However, when we collapsed the disorder responses and deleted the sleeping item, the test information was significantly decreased (it changed from 12.2 to 6.6).

### Limitations

Several limitations of our study are worth noting. First, although the source of cases was from different departments and included both outpatients and inpatients, patients with back pain from the rheumatology departments were not included our study. Whether the items of ODI still fit the model and could precisely assess the patient disability for this population is not known. Further work is suggested to explore the psychometric properties for patients visiting the rheumatology department. Second, there may be other variables that affect responses with DIF. DIF analysis has been used to detect item equivalence across age, gender, racial, cultural, treatment groups, and with different administration models [37-41]. The DIF may be presented in different samples that live in community or in institutions. The DIF may also present in different administration methods, such as assessment by self-administration or interview; the problems may particularly occur in self-administration of the questionnaire for elderly patients, those with less education, and those with cognitive problems. The issue of DIF is essential to improve the generalizability of the test. Deleting the DIF item can improve the psychometric properties of the test; however, if the item is important for clinical application, it can demonstrate that the important situation is rather different in groups. Providing a different version to suit the special groups may also be useful in clinical practice. Finally, many cutoff points of statistics in Rasch analysis are inconsistent; therefore, more consensus studies in the future will be helpful in further research of Rasch analysis.

## Conclusions

The ODI version 2.1 is a unidimensional questionnaire with high reliability, and well estimates the disability level of back pain without skewing toward the lower or higher levels of disability. The ODI is a simple disability scale that uses only 10 items to measure the disability level, and the order of item difficulty could be used as a rule of progressive management program. The item difficulty showed that traveling activity is easily limited for patients with back pain. The results suggest the most precise estimation of functional limitation focuses about the range of −2.3 to 2.3 logits disability level, equal to the range of 5 to 45 raw scores of the ODI.

## Competing interests

The authors declare that they have no competing interests.

## Authors’ contributions

YJL and YML were the principal and co-principal investigators of the study, designed the study, acquired funding, were responsible for data collection, and wrote the manuscript. YYW and CLH were responsible for the methodological issue, contributed the statistical analyses and drafted the manuscript. YML, CLL, SLH and KIC contributed to the recruitment and diagnostic issues of the clinical sample and were responsible for the overall design of the study. All authors read and approved the final manuscript.
